# Prevalence and association of refractive anisometropia with near work habits among young schoolchildren: The evidence from a population-based study

**DOI:** 10.1371/journal.pone.0173519

**Published:** 2017-03-08

**Authors:** Chia-Wei Lee, Shao-You Fang, Der-Chong Tsai, Nicole Huang, Chih-Chien Hsu, Shing-Yi Chen, Allen Wen-Hsiang Chiu, Catherine Jui-Ling Liu

**Affiliations:** 1 Department of Ophthalmology, Taipei Veterans General Hospital, Taipei, Taiwan; 2 Children and Family Research Center, National Taiwan University, Taipei, Taiwan; 3 National Yang-Ming University School of Medicine, Taipei, Taiwan; 4 Department of Ophthalmology, National Yang-Ming University Hospital, Yilan, Taiwan; 5 Institute of Hospital and Health Care Administration, National Yang-Ming University, Taipei, Taiwan; 6 Institute of Clinical Medicine, National Yang-Ming University, Taipei, Taiwan; 7 Department of Health, Taipei City Government, Taipei, Taiwan; National Eye Institute, UNITED STATES

## Abstract

**Background:**

Lifestyle behaviour may play a role in refractive error among children, but the association between near work habits and refractive anisometropia remains unclear.

**Methods:**

We estimated the prevalence of refractive anisometropia and examined its association with near work activities among 23,114 children in the Myopia Investigation Study in Taipei who were grade 2 elementary school students at baseline in 2013 and 2014. Baseline data on demographics, medical history, parental history and near work habits were collected by parent-administered questionnaire survey. Refractive status was determined by cycloplegic autorefraction. Refractive anisometropia was defined as the spherical equivalent difference ≥ 1.0 diopter between eyes.

**Results:**

The prevalence of refractive anisometropia was 5.3% (95% confidence interval [CI], 5.0% to 5.6%). The prevalence and severity of refractive anisometropia increased with both myopic and hyperopic refractive error. Multivariate logistic regression analysis revealed that refractive anisometropia was significantly associated with myopia (odds ratio [OR], 2.98; 95% CI, 2.53–3.51), hyperopia (OR, 2.37; 95% CI, 1.98–2.83), degree of astigmatism (OR, 1.005; 95% CI, 1.005–1.006), amblyopia (OR, 2.54; 95% CI, 2.06–3.12), male gender (OR, 0.88; 95% CI, 0.78–0.99) and senior high school level of maternal education (OR, 0.69; 95% CI, 0.52–0.92). Though anisometropic children were more likely to spend more time on near work (crude OR, 1.15; 95% CI, 1.02–1.29) and to have less eye-to-object distance in doing near work (crude OR, 1.15; 95% CI, 1.01–1.30), these associations became insignificant after additional adjustment for ocular, demographic and parental factors.

**Conclusions:**

The present study provides large-scale, population-based evidence showing no independent association between refractive anisometropia and near work habits, though myopia is associated with refractive anisometropia.

## Introduction

Refractive anisometropia, a between-eye difference in ocular refraction, is of great clinical interest because it is a well-known amblyogenic factor in children. Early detection of refractive anisometropia in children with timely intervention could prevent permanent impairment in binocular vision and stereopsis. Refractive anisometropia represents a unique condition in that the fellow eyes of an individual with presumably similar sociodemographic, environmental, and genetic influences can have asymmetric ocular growth. Thus, investigation of refractive anisometropia among schoolchildren is helpful to understand the development of refractive error.

The structural basis of refractive anisometropia is attributed to the interocular difference in axial length [1]. It has been reported that asymmetry in visual experience during childhood has the potential to alter axial growth [2, 3, 4]. Near work activities can generate transient axial elongation [5, 6]. Woodman et al. [7] reported greater increases in magnitude of transient axial elongation and time for recovery in myopic subjects compared with emmetropic subjects. Common hypothesis of mechanisms leading to axial elongation include biomechanical forces generated during accommodation or convergence [5, 6, 7], a human visual system response to a hyperopic defocused image that induces an adjustment in the position of the retina to compensate image blur [8], or a combination of the two. Given that the two eyes of an individual may have different refractive status at baseline, with stimuli of unequal convergent or accommodative demand during near work tasks, these interocular differences in visual experience may potentially induce asymmetric axial length elongation and result in refractive anisometropia.

Despite a number of studies describing the ocular structure, demographic and refractive status of subjects with anisometropia [9–20], epidemiological evidence on the relationship between refractive anisometropia and lifestyle among school-aged children is generally insufficient [19]. Since lifestyle is a readily modifiable factor and has been well documented to be associated with childhood myopia [21,22], it is worth exploring whether near work habits contribute to the development of refractive anisometropia. In the present study, we analysed the baseline data of the Myopia Investigation Study in Taipei (MIT) and aimed to report the prevalence of refractive anisometropia and evaluate the association between near work habits and refractive anisometropia among young schoolchildren.

## Materials and methods

### Design and subjects

The MIT, a citywide, population-based cohort study, is designed to investigate the refractive status in schoolchildren for 3 consecutive years. The study design, methods and rationale have been previously described elsewhere [23]. In brief, since school year 2013, all schoolchildren of second grade in Taipei City were invited to participate in this study. Written informed consent had been obtained from parents of the participants before being enrolled. MIT-associated medical facilities provided eye examinations for eligible schoolchildren each semester for 3 consecutive years. The baseline results of the first two school-year cohorts (enrolled in 2013 and 2014, respectively) were reported here. All examinations were requested and monitored to be compliant with the standard operation procedure set by the MIT committee that supervised the quality of study execution. The protocol and consent procedure were approved by the Institutional Review Board of the Taipei City Hospital (TCHIRB-1020501) and all procedures adhered to the Declaration of Helsinki.

### Measurement of refractive status

The eye examination for the MIT participants include slit lamp examination and the measurement of visual acuity (unaided and best-corrected) and refractive status (before and after cycloplegia). Three kinds of cycloplegic eye drops were approved for use in the MIT, including Cyclogyl (1% cyclopentolate), Mydriacyl (1% tropicamide), and Mydrin-P (0.5% phenylephrine hydrochloride/0.5% tropicamide). Among these three drugs, each hospital or clinic could use only one kind of cycloplegic eye drop to perform cycloplegia for the MIT participants. Two drops of the cycloplegic drug were administered 10 minutes apart. Thirty minutes after the second drop of the cycloplegic drug, a penlight was used to check the pupil light reflex. If the pupil still responded to penlight, an additional 10-minunte wait was required before cycloplegic refraction. Refractive status was determined by the average of three consecutive autorefraction measurements. The spherical equivalent (SE) of the refractive error was calculated as: the spherical power plus half the magnitude of the cylinder power. Only the eye with less SE (less positive or more negative refractive error) in each child was used for defining the baseline SE and the degree of astigmatism.

Similar to previous epidemiological studies [24], we defined myopia as ≤-0.5 D SE (mild myopia [-0.5 D ≥ SE > -3.0 D]; moderated myopia [-3.0 D ≥ SE > -6.0 D] and high myopia [-6.0 D ≥ SE]), emmetropia as +0.5 D > SE > -0.5 D, hyperopia as ≥ +0.5 D SE (mild hyperopia [+2.0 D > SE ≥ +0.5 D]; moderate hyperopia [+5.0 D > SE ≥ +2.0 D] and high hyperopia [SE ≥ +5.0 D]), astigmatism as cylinder power ≤ −1.0D and refractive anisometropia as the SE difference ≥ 1.0 D between eyes. The level of refractive anisometropia severity was categorized into mild (SE difference ≥ 1.0 D and < 2.0 D), moderate (SE difference ≥ 2.0 D and <3.0 D), and severe (SE difference ≥ 3.0 D). The proportion of different severity levels of refractive anisometropia by the extent of SE in less ametropic eye was calculated. Subjects were categorized according to the SE in less ametropic eye to minimize the potential effect of spurious association between anisometropia and refractive error [25].

### Assessment of potential risk factors

We hypothesized that the potential risk factor for anisometropia may include ocular, demographic, and parental factors and near work habits. The data of these potential risk factors were obtained from parent-administered questionnaire surveys covering demographics, medical history, parental history, time spent on near work and outdoor activities, reading habits, and eye-care seeking behavior. The MIT parent questionnaire was composed of closed-ended questions with two option (yes/no) responses or a list of ordered choices. Respondents were asked to check the choice they felt was the most proper. Questions regarding near work habits and analyzed in the present study included age of near work initiation, eye-to-object distance, use of computer or mobile devices, average daily quantity of near work activities, including reading, writing, painting, playing instruments, watching television, and playing computer/video games, the presence of adequate rest, as well as attending an after-school tutorial program.

### Statistical analysis

The data of participants enrolled in 2013 or 2014 who had completed the cycloplegic autorefraction measurements and responded to the survey were analysed. All data were expressed as mean± SD or percentage. The differences between groups in terms of refractive status, sociodemographic characteristics, parental myopia status, and near work activities were compared with Chi-square tests or independent t-tests as appropriate. To examine the potential association between ocular, demographic and parental factors, near work activities, and refractive anisometropia, multivariate logistic regression analyses were conducted with the presence of refractive anisometropia as the dependent variable. To estimate how, and to what extent, these factors influenced refractive anisometropia, a hierarchical logistic regression was performed. Ocular, demographic, and parental factors and near work activities were entered block-wise into models. The estimated risk of refractive anisometropia was separately assessed using three models with different arrays of independent variables. Model 1 consisted of ocular factors (baseline refractive status, presence of amblyopia, and degree of astigmatism), and Model 2 combined ocular factors in Model 1 with demographic and parental factors (gender, region of residence, maternal education level, and parental myopia status). In the final model (Model 3), all factors in Model 1 and Model 2 as well as near work habits (age of near work initiation, average time spent on near work per day, eye-to-object distance in doing near work, 10-minute rest period after 30 minutes of near work, use of computer or mobile device in the past year, and attending an after-school tutorial program) were included. The values of adjusted pseudo-R^2^ and Akaike information criterion (AIC) of each model were also calculated. The pseudo-R^2^ value in the logistic regression model is helpful to understand how much of the variance is accounted for by the independent variables. The AIC value is used for model selection. The model with a lower AIC value is a better fit for the data and loses less information. Furthermore, to check the possible association of near work habits with severe refractive anisometropia, multivariate logistic regression analysis was also conducted with the presence of severe refractive anisometropia as the dependent variable for the sensitivity analysis. It has been reported that more time spent on near work activities is a risk factor for childhood myopia[26]. Hence, subgroup analyses were conducted to explore the association between near work habits and refractive anisometropia separately among myopic and hyperopic subjects. Statistical analysis was performed using Statistical Analysis System Software (Statistical Analysis System Software V9.3, SAS Institute, Cary, North Carolina, USA). Multicollinearity diagnostics were assessed on the basis of the suggestions in the SAS logistic regression model. All statistical tests were two-sided, and p < 0.05 was considered statistically significant.

## Results

**Fig 1:**
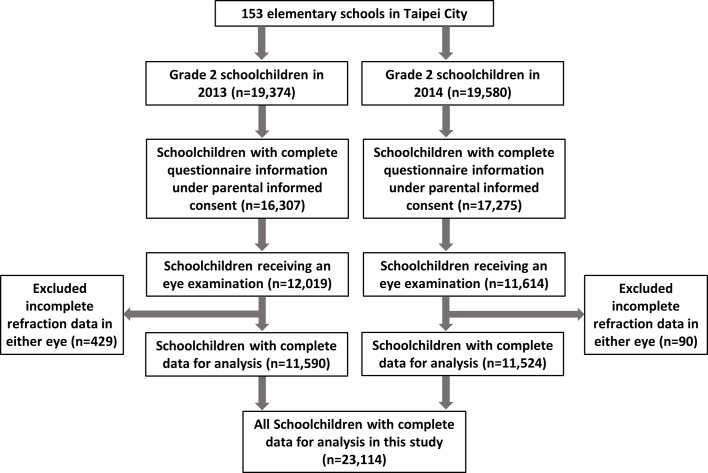
A flowchart showing the ascertainment process of participants of the study.

**Fig 2:**
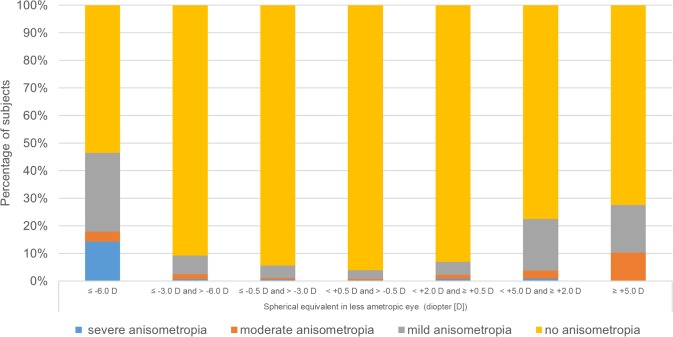
The proportion of different severity levels of refractive anisometropia by refractive groups.

A total of 23,114 subject (11,090 [48%] female) were finally recruited into the statistical analysis. Fig 1 shows a flow chart of the ascertainment process of the subjects participating in each stage of the study. Of these participants, 1,218 (prevalence rate, 5.3%; 95% confidence interval [CI], 5.0%-5.6%) were found to have refractive anisometropia. Of these 1,218 anisometropic subjects, the mean SE difference between eyes was 1.70 ±1.16D (range 1-14D), and 940 (77.2%), 170 (14.0%) and 108 (8.9%) were mild, moderate and severe level of refractive anisometropia, respectively. The distributions of refractive status, demographic characteristics, parental history and near work activities for subjects with and without anisometropia are shown in Table 1. The mean baseline SE of all participants was -0.28 ±1.23D. Children with refractive anisometropia had more amount of myopic SE than those without anisometropia (-0.86 ±2.36D vs. -0.24 ±1.13D, p<0.001). Those with refractive anisometropia also had more severe astigmatism and were more likely to have amblyopia. As illustrated in Fig 2, the distributions of the prevalence and severity of refractive anisometropia by the extent of SE in less ametropic eye showed a V-shaped trend. That is, refractive anisometropia was more prevalent in the myopic and hyperopic groups (8.32% and 5.86%, respectively) than in the emmetropic group (2.27%). The proportion of anisometropia ≥ 2.0 D was also higher among myopes (1.80%) and hyperopes (1.52%) when compared to emmetropes (0.50%).

**Table 1:** Characteristics of Participants.

	Total (n = 23,114)		Without refractive anisometropia (n = 21,896)		With refractive anisometropia (n = 1,218)		P value
**Refractive status**, n(%)							<0.001
Hyperopia	5,543	(24.0)	5,218	(23.8)	325	(26.7)	
Myopia	8,153	(35.3)	7,474	(34.1)	679	(55.8)	
Emmetropia	9,418	(40.7)	9,204	(42.0)	214	(17.5)	
**Amblyopia**, n(%)							<0.001
Yes	808	(3.5)	660	(3.0)	148	(12.2)	
No	22,306	(96.5)	21,236	(97.0)	1,070	(87.8)	
**Astigmatism**, mean (SD)	-0.79	(0.78)	-0.75	(0.73)	-1.39	(1.21)	<0.001
**Gender**, n(%)							0.092
Male	12,024	(52.0)	11,419	(52.2)	605	(49.7)	
Female	11,090	(48.0)	10,477	(47.8)	613	(50.3)	
**Area of residence**, n(%)							0.602
Urban	11,763	(50.9)	11,152	(50.9)	611	(50.2)	
Suburban	11,351	(49.1)	10,744	(49.1)	607	(49.8)	
**Maternal education level**, n(%)							0.011
Junior high school or less	890	(3.9)	822	(3.8)	68	(5.6)	
Senior high school	10,295	(44.5)	9,766	(44.6)	529	(43.4)	
College or more	11,219	(48.5)	10,630	(48.5)	589	(48.4)	
Unknown	710	(3.1)	678	(3.1)	32	(2.6)	
**Parental myopia**, n(%)							0.668
None	1,953	(8.5)	1,840	(8.4)	113	(9.3)	
Either myopic	7,304	(31.6)	6,919	(31.6)	385	(31.6)	
Both myopic	13,136	(56.8)	12,457	(56.9)	679	(55.8)	
Unknown	721	(3.1)	680	(3.1)	41	(3.3)	
**Age when starting near work**, n(%)							0.092
<6 years	20,341	(88.0)	19,261	(88.0)	1,080	(88.7)	
≥6 years	2,241	(9.7)	2,139	(9.8)	102	(8.4)	
Unknown	532	(2.3)	496	(2.2)	36	(2.9)	
**Time spent on near work**, n(%)							0.070
<2 hours per day	13,620	(58.9)	12,940	(59.1)	680	(55.8)	
≥2 hours per day	8,829	(38.2)	8,326	(38.0)	503	(41.3)	
Unknown	665	(2.9)	630	(2.9)	35	(2.9)	
**Distance from near work**, n(%)							0.069
≥30 centimeters	11,467	(49.6)	10,902	(49.8)	565	(46.4)	
<30 centimeters	8,666	(37.5)	8,180	(37.4)	486	(39.9)	
Unknown	2,981	(12.9)	2,814	(12.8)	167	(13.7)	
**10-minute rest period after 30 minutes of near work**, n(%)							0.221
Yes	9,513	(41.2)	9,026	(41.2)	487	(40.0)	
No	9,236	(40.0)	8,758	(40.0)	478	(39.2)	
Unknown	4,365	(18.8)	4,112	(18.8)	253	(20.8)	
**Use of cellphones, computers, or computer tablets in the past year**, n(%)							0.970
Yes	20,131	(87.1)	19,073	(87.1)	1,058	(86.9)	
No	2,254	(9.8)	2,133	(9.7)	121	(9.9)	
Unknown	729	(3.1)	690	(3.2)	39	(3.2)	
**Attending an after-school tutorial program**, n(%)							0.718
Yes	16,514	(71.5)	15,656	(71.5)	858	(70.5)	
No	5,921	(25.6)	5,599	(25.6)	322	(26.4)	
Unknown	679	(2.9)	641	(2.9)	38	(3.1)	

SE, spherical equivalent; SD, standard deviation.

Table 2 lists the results of multiple logistic regression analysis. In Model 1, refractive anisometropia was significantly associated with SE at baseline (myopic SE: odds ratio [OR], 2.89; 95% CI, 2.46–3.40; hyperopic SE: OR, 2.42; 95% CI, 2.02–2.89), amblyopia (OR, 2.54; 95% CI, 2.07–3.12), and astigmatism (OR, 1.005; 95% CI, 1.005–1.006). These associations between refractive anisometropia and ocular factors remained significant across all three models. In Model 2, when demographic and parental factors were included, male gender (OR, 0.88, 95% CI, 0.78–0.99) and higher maternal education level (senior high school level: OR, 0.71; 95% CI, 0.53–0.94) were also found to have significant associations with refractive anisometropia. However, there was no significant association between refractive anisometropia and any near work activities in Model 3. These findings remained consistent when the sensitivity analysis was performed by defining severe refractive anisometropia as a dependent variable. The explanatory power for refractive anisometropia slightly increased from Model 1 (adjusted pseudo-R^2^, 0.1012) to Model 3 (adjusted pseudo-R^2^, 0.1059), whereas the increment between Model 2 and Model 3 (Δpseudo-R^2^ = 0.0019) was smaller than that between Model 1 and Model 2 (Δpseudo-R^2^ = 0.0028). Among all 3 models, Model 2 had the lowest AIC value (8738.672).

**Table 2:** Multivariate logistic regression analysis of refractive anisometropia among all participants (n = 23,114).

				Model 1			Model 2			Model 3		
	Crude OR	*P* value	95% CI	Adjusted OR	*P* value	95% CI	Adjusted OR	*P* value	95% CI	Adjusted OR	*P* value	95% CI
**Refractive status**												
Hyperopia	2.68	<0.001	2.25–3.19	2.42	<0.001	2.02–2.89	2.37	<0.001	1.98–2.83	2.37	<0.001	1.98–2.83
Myopia	3.91	<0.001	3.34–4.57	2.89	<0.001	2.46–3.40	2.97	<0.001	2.53–3.50	2.98	<0.001	2.53–3.51
Emmetropia	1.00			1.00			1.00			1.00		
**Astigmatism** (diopter)	1.006	<0.001	1.006–1.007	1.005	<0.001	1.005–1.006	1.005	<0.001	1.005–1.006	1.005	<0.001	1.005–1.006
**Amblyopia**												
Yes	4.45	<0.001	3.69–5.37	2.54	<0.001	2.07–3.12	2.55	<0.001	2.08–3.14	2.54	<0.001	2.06–3.12
No	1.00			1.00			1.00			1.00		
**Gender**												
Male	0.91	0.092	0.81–1.02				0.88	0.034	0.78–0.99	0.88	0.039	0.78–0.99
Female	1.00						1.00			1.00		
**Area of residence**												
Urban	0.97	0.602	0.86–1.09				0.98	0.699	0.87–1.10	0.97	0.669	0.87–1.10
Suburban	1.00						1.00			1.00		
**Maternal education level**												
Junior high school or less	1.00						1.00			1.00		
Senior high school	0.66	0.002	0.50–0.85				0.71	0.017	0.53–0.94	0.69	0.011	0.52–0.92
College or more	0.67	0.003	0.52–0.87				0.83	0.194	0.62–1.10	0.80	0.131	0.59–1.07
**Parental myopia**												
None of both	1.00						1.00			1.00		
Either myopic	0.91	0.371	0.73–1.12				0.93	0.505	0.74–1.16	0.92	0.461	0.73–1.15
Both myopic	0.89	0.254	0.72–1.09				0.81	0.073	0.65–1.02	0.80	0.058	0.64–1.01
**Age when starting near work**												
<6 years	1.18	0.127	0.96–1.45							1.16	0.168	0.94–1.44
≥6 years	1.00									1.00		
**Time spent on near work**												
<2 hours per day	1.00									1.00		
≥2 hours per day	1.15	0.021	1.02–1.29							1.10	0.132	0.97–1.25
**Distance from near work**												
≥30 centimeters	1.00									1.00		
<30 centimeters	1.15	0.032	1.01–1.30							1.03	0.675	0.90–1.17
**10-minute rest period after 30 minutes of near work**												
Yes	1.00									1.00		
No	1.01	0.862	0.89–1.15							0.93	0.289	0.81–1.07
**Use of cellphones, computers, or computer tablets in the past year**												
Yes	0.98	0.819	0.81–1.19							1.06	0.597	0.86–1.29
No	1.00									1.00		
**Attending an after-school tutorial program**												
Yes	0.95	0.473	0.84–1.09							0.90	0.141	0.79–1.04
No	1.00									1.00		
**Psuedo-R**^**2**^						0.1012	0.1040					0.1059
**Akaike information criterion**						8745.589	8738.672					8747.519

OR, odds ratio; CI, confidence interval.

Table 3 and Table 4 show the results of subgroup regression analysis restricted to myopic and hyperopic schoolchildren, respectively. Among 8,153 myopic schoolchildren, refractive anisometropia was independently associated with high myopia (OR, 14.67; 95% CI, 8.67–24.82), moderate myopia (OR, 1.97; 95% CI, 1.55–2.51), amblyopia (OR, 1.43; 95% CI, 1.05–1.95), astigmatism (OR, 1.004; 95% CI, 1.003–1.005), and higher maternal education level (senior high school level: OR, 0.54; 95% CI, 0.37–0.80; college level or more: OR, 0.63; 95% CI, 0.42–0.94). Similarly, the risk factors of refractive anisometropia among hyperopic schoolchildren included high hyperopia (OR, 3.89; 95% CI, 1.69–8.92), moderate hyperopia (OR, 2.90; 95% CI, 2.10–4.01), amblyopia (OR, 4.01; 95% CI, 2.79–5.77), and astigmatism (OR, 1.004; 95% CI, 1.003–1.005). Of note, no significant association between refractive anisometropia and near work activities was found in both subgroup analyses. Multicollinearity diagnostics were examined and variance inflation factors between any variables were less than 1.3 in all models.

**Table 3:** Multivariate logistic regression analysis of refractive anisometropia among myopic participants (n = 8,153).

Characteristics	Crude OR	*P* value	95% CI	Adjusted OR	*P* value	95% CI
**Baseline SE**						
SE ≤ -6.0D	23.54	<0.001	14.32–38.68	14.67	<0.001	8.67–24.82
-6.0D < SE ≤ -3.0D	2.62	<0.001	2.09–3.29	1.97	<0.001	1.55–2.51
-3.0D < SE ≤ -0.5D	1.00			1.00		
**Astigmatism** (diopter)	1.005	<0.001	1.004–1.005	1.004	<0.001	1.003–1.005
**Amblyopia**						
Yes	2.62	<0.001	2.00–3.45	1.43	0.024	1.05–1.95
No	1.00			1.00		
**Gender**						
Male	0.85	0.048	0.73–0.99	0.86	0.079	0.73–1.02
Female	1.00			1.00		
**Area of residence**						
Urban	1.04	0.623	0.89–1.22	1.05	0.553	0.89–1.24
Suburban	1.00			1.00		
**Maternal education level**						
Junior high school or less	1.00			1.00		
Senior high school	0.53	<0.001	0.37–0.75	0.54	0.002	0.37–0.80
College or more	0.55	0.001	0.39–0.78	0.63	0.024	0.42–0.94
**Parental myopia**						
None of both	1.00			1.00		
Either myopic	0.74	0.087	0.52–1.05	0.86	0.420	0.59–1.25
Both myopic	0.80	0.177	0.58–1.11	0.85	0.396	0.59–1.23
**Age when starting near work**						
<6 years	0.99	0.945	0.75–1.31	1.00	0.978	0.75–1.34
≥6 years	1.00			1.00		
**Time spent on near work**						
<2 hours per day	1.00			1.00		
≥2 hours per day	1.01	0.878	0.86–1.19	1.02	0.813	0.86–1.21
**Distance from near work**						
≥30 centimeters	1.00			1.00		
<30 centimeters	1.12	0.177	0.95–1.33	1.03	0.774	0.86–1.23
**10-minute rest period after 30 minutes of near work**						
Yes	1.00			1.00		
No	0.96	0.670	0.81–1.15	0.93	0.436	0.77–1.12
**Use of cellphones, computers, or computer tablets in the past year**						
Yes	0.87	0.255	0.68–1.11	0.90	0.403	0.70–1.16
No	1.00			1.00		
**Attending an after-school tutorial program**						
Yes	0.90	0.254	0.75–1.08	0.88	0.186	0.73–1.06
No	1.00			1.00		

OR, odds ratio; CI, confidence interval; SE, spherical equivalent.

**Table 4:** Multivariate logistic regression analysis of refractive anisometropia among hyperopic participants (n = 5,543).

Characteristics	Crude OR	*P* value	95% CI	Adjusted OR	*P* value	95% CI
**Baseline SE**						
SE ≥ 5.0D	6.88	<0.001	3.19–14.84	3.89	0.001	1.69–8.92
2.0D ≤ SE < 5.0D	4.85	<0.001	3.62–6.49	2.90	<0.001	2.10–4.01
0.5D ≤ SE < 2.0D	1.00			1.00		
**Astigmatism** (diopter)	1.006	<0.001	1.005–1.008	1.004	<0.001	1.003–1.005
**Amblyopia**						
Yes	6.78	<0.001	4.90–9.38	4.01	<0.001	2.79–5.77
No	1.00			1.00		
**Gender**						
Male	1.03	0.789	0.82–1.29	1.06	0.618	0.84–1.34
Female	1.00			1.00		
**Area of residence**						
Urban	0.83	0.109	0.67–1.04	0.84	0.146	0.67–1.06
Suburban	1.00			1.00		
**Maternal education level**						
Junior high school or less	1.00			1.00		
Senior high school	0.66	0.108	0.40–1.10	0.67	0.155	0.39–1.16
College or more	0.62	0.056	0.37–1.01	0.73	0.263	0.42–1.27
**Parental myopia**						
None of both	1.00			1.00		
Either myopic	1.12	0.542	0.78–1.60	1.17	0.407	0.80–1.71
Both myopic	0.84	0.337	0.59–1.20	0.97	0.884	0.66–1.44
**Age when starting near work**						
<6 years	1.32	0.168	0.89–1.97	1.25	0.293	0.83–1.88
≥6 years	1.00			1.00		
**Time spent on near work**						
<2 hours per day	1.00			1.00		
≥2 hours per day	1.20	0.127	0.95–1.51	1.11	0.398	0.87–1.42
**Distance from near work**						
≥30 centimeters	1.00			1.00		
<30 centimeters	0.99	0.909	0.77–1.26	0.87	0.282	0.67–1.12
**10-minute rest period after 30 minutes of near work**						
Yes	1.00			1.00		
No	1.14	0.308	0.89–1.46	1.11	0.427	0.85–1.45
**Use of cellphones, computers, or computer tablets in the past year**						
Yes	1.23	0.344	0.81–1.86	1.34	0.200	0.86–2.08
No	1.00			1.00		
**Attending an after-school tutorial program**						
Yes	1.07	0.589	0.83–1.38	1.03	0.820	0.79–1.34
No	1.00			1.00		

OR, odds ratio; CI, confidence interval; SE, spherical equivalent.

## Discussion

From the baseline data of this large-scale, population-based study, we observed that the prevalence of refractive anisometropia was 5.3% among 23,114 predominantly 8-year-old schoolchildren in a metropolitan city in East Asia and refractive anisometropia was associated with baseline refractive status, astigmatism, presence of amblyopia, gender and maternal education level. None of the factors regarding near work activities was found to be a risk factor for refractive anisometropia. To our knowledge, the present study provides the first population-based data on the epidemiology of refractive anisometropia among schoolchildren in Taiwan.

The main results of epidemiological studies on prevalence of refractive anisometropia among children and adolescents are summarized in Table 5. Even using the same definition of refractive anisometropia (SE difference between eyes ≥ 1.0 D), there is significant variation in anisometropia prevalence across studies differing in terms of age, country and ethnicity [11–19]. In Australia, Huynh et al revealed a prevalence rate of 1.6% among 1,765 predominantly 6-year-old children [11]. The prevalence rates were reported by O’Donoghue et al. to be 8.5% and 9.4% for 6- to 7-year-old and 12- to 13-year-old children from Northern Ireland [18]. Tong et al. reported a prevalence of 3.6% in Singapore school children aged 7 to 9 years (2.7%, 3.0% and 5.8% for 7-, 8-, and 9-year-olds, respectively) [12]. In a large-scale school-based study in Eastern China, Hu et al found that 7% of 6,025 schoolchildren aged 4 to 18 years had refractive anisometropia [19]. The prevalence of refractive anisometropia among grade 2 schoolchildren in our study was similar to and between that in East Asian countries [12,19]. In addition, our results were higher than those for preschool or 6-year-old children populations [11,13,14,17]. Increasing age has been found to be significantly associated with the prevalence of refractive anisometropia [12,16,19]. The studied age range difference may partially account for the difference in the refractive anisometropia prevalence between our study and others.

**Table 5:** Epidemiological studies on the prevalence of refractive anisometropia among children.

Study (year)	Number of participant and area	Study design	Ethnicity	Age	Prevalence of anisometropia
**Huynh** et al. [11] (2006)	n = 1765; Australia	Population-based	European white (63.6%), East Asian (17.1%)	6 years	1.6%
**Tong** et al. [12] (2006)	n = 1979; Singapore	Population-based	East Asian and South Asian	7 to 9 years	3.6%
**Giordano** et al. [13] (2009)	n = 2298; USA	Population-based	White and African-American	6 to 72 months	5.0% (white), 4.3% (African-American)
**Borchert** et al. [14] (2010)	n = 6024; USA	Population-based	Hispanic and African-American	6 to 72 months	4.0% (Hispanics), 4.2% (African-American)
**Yekta** et al. [15] (2010)	n = 1872; Iran	Population-based	Iranian	7 to 15 years	2.6%
**Deng** et al. [16] (2012)	n = 1120; USA	Longitudinal	White	6 months, 5 years and 12 to 15 years	1.96% at 6 months, 1.27% at 5 years, 5.7% at 12 to 15 years
**Afsari** et al. [17] (2013)	n = 2090; Australia	Population-based	European-Caucasian (46.9%), Asian (33.4%), Others (19.6%)	6 to 72 months	2.7%
**O’Donoghue** et al. [18] (2013)	n = 1053; Northern Ireland	Population-based	European-Caucasian	6–7 and 12–13 years	8.5% at 6–7 years, 9.4% at 12–13 years
**Hu** et al. [19] (2016)	n = 6025; China	School-based	East Asian	4 to 18 years	7.0%
**The present study**	n = 23,114; Taiwan	Population-based	East Asian	8 years	5.3%

Near work activities have been found to be associated with childhood myopia in previous epidemiological studies [27–30]. However, interocular difference in accommodative responsivity to near work activities and the association between near work and refractive anisometropia are still poorly understood among schoolchildren. Lin et al. examined the short term changes of ocular biometric characteristics during near work activities in anisometropic individuals and found that more myopic eyes exhibited significantly increased near work-induced transient myopia magnitudes and increased decay times as compared to less myopic eyes in approximately two-thirds of the subjects. Nevertheless, those ocular biometric changes were transient phenomena instead of permanent unequal axial elongation [31]. It has been reported that accommodative response is correlated with ciliary muscle thickness and thicker ciliary body measurements are associated with myopia [32,33]. However, a recent anisometropia study found no significant difference in ciliary muscle thickness between the two eyes among anisometropic subjects [34].

Recently, Hu et al. reported that more time spent indoors reading or writing was significantly associated with higher prevalence of refractive anisometropia (OR, 1.12; 95% CI, 1.04–1.20) and higher amount of refractive anisometropia and anisomyopia (ß: 0.04, P < 0.001; ß: 0.03, P = 0.01; respectively) in the multivariate models [19]. In the present study, compared to the isometropic schoolchildren, those with refractive anisometropia were more likely to spend 2 hours or more on near work per day (38.0% vs 41.3%; crude OR, 1.15; 95% CI, 1.02–1.29) and to keep less than 30 cm between the eyes and object in doing near work (37.4% vs 39.9%; crude OR, 1.15; 95% CI, 1.01–1.30). However, after controlling for other potential confounders, no factor regarding the near work habits was found to have independent association with refractive anisometropia in the full model (Model 3). Besides, a small difference in the values of adjusted pseudo-R^2^ between Model 2 (0.1040) and Model 3 (0.1059) indicated a poor explanatory power attributable to the near work-related variables added in Model 3. Furthermore, Model 2, including ocular, demographic and parental factors as independent variables, had the lowest AIC value and was the best fit among all 3 models. Subgroup analysis also showed no association between near work habits and refractive anisometropia among both myopic and hyperopic schoolchildren. In the questionnaire survey of our study, time spent on near work activities was defined as the average daily quantity of all kinds of near work, including reading, writing, painting, playing instruments and playing computer/video games over the past year [23]. By contrast, only time spent indoors reading or writing, not time spent indoors playing handheld computer games nor total time indoors, entered the final multivariate models in the study by Hu et al. The difference in study design and methodology between studies may partially explain the disparity regarding the association between near work and refractive anisometropia.

Consistent with previous literature [16,25], the risks of having refractive anisometropia were higher in myopes and hyperopes compared with emmetropes among young schoolchildren in our study. As illustrated in Fig 1, the distributions of the prevalence and severity of refractive anisometropia by refractive groups also showed a V-shaped trend: an increase in the prevalence and degree of refractive anisometropia with refractive errors diverging from emmetropia in both myopic and hyperopic directions. Interestingly, Qin et al.[25] and Guzowski et al. [35] also observed similar distribution patterns between anisometropia and ametropia among older adults. The pathogenesis of refractive anisometropia is considered to be different between children and elder populations. The age-related ocular pathology, such as unequal progress of cataract, may play an important role in older adult’s anisometropia.

The present study has some strengths. Due to the well-executed compulsory education system in Taiwan, the MIT study, for the purpose of identifying every undiagnosed myope in early childhood, invited all grade 2 schoolchildren in Taipei City to participate with the help of school authorities. This design makes it as one of the largest epidemiological studies of anisometropia among schoolchildren and minimizes potential selection bias occurring in the sampling procedure. In addition, the high number of participants from the population gives the study greater power to detect differences.

Several limitations of the present study should be noted. First, similar to a cross-sectional study design, only baseline data of the MIT cohorts was analyzed in the present study and it is hard to differentiate cause and effect from simple association. Second, near work activities of schoolchildren were assessed based on parent-completed questionnaire surveys rather than direct measurement. Therefore, our analysis is subject to recall bias. The questionnaire, while practical, may not be the most accurate tool to evaluate the quantity of near work activities. Parents whose children have had myopia may be more likely than parents of non-myopic children to remember details of myopiogenic behaviors. However, no independent association between near work activities and refractive anisometropia was found in both myopic and hyperopic groups in our study. Third, ocular biometric measurements were not carried out in the MIT study. Therefore, the data regarding axial length was not available. Finally, generalizability of anisometropia data in MIT to other ethnic populations is a concern.

## Conclusion

The present study provides large-scale, population-based evidence regarding the estimation of prevalence and risk factors for refractive anisometropia among young schoolchildren in Taipei City. Though anisometropic children were more likely to spend more time on near work and to have less eye-to-object distance in doing near work, these associations became insignificant after additional adjustment for ocular, demographic and parental factors. The impact of near work habits on the development of refractive anisometropia still warrants further large-scale cohort studies among schoolchildren.

## Supporting information

S1 Table Survey questions and responses in the questionnaire. (DOC)

## References

[1] Sorsby A, Leary G, Joan Richards M. The optical components in anisometropia. Vision Research. 1962;2: 43–51.

[2] Gee SS, Tabbara KF. Increase in ocular axial length in patients with corneal opacification. Ophthalmology. 1988;95: 1276–1278. 3062539 10.1016/s0161-6420(88)33035-6

[3] Von Noorden GK, Lewis RA. Ocular axial length in unilateral congenital cataracts and blepharoptosis. Invest Ophthalmol Vis Sci. 1987; 28: 750–752. 3557880

[4] Swarbrick H, Alharbi A, Watt K, Lum E, Kang P. Myopia control during orthokeratology Lens wear in children using a novel study design. Ophthalmology. 2015;122: 620–630. 10.1016/j.ophtha.2014.09.028 25439432

[5] Ghosh A, Collins M, Read S, Davis B, Chatterjee P. Axial elongation associated with biomechanical factors during near work. Optometry and Vision Science. 2014;91: 322–329. 10.1097/OPX.0000000000000166 24413276

[6] Mallen EA, Kashyap P, Hampson KM. Transient axial length change during the accommodation response in young adults. Invest Ophthalmol Vis Sci. 2006;47: 1251–1254. 10.1167/iovs.05-1086 16505066

[7] Woodman E, Read S, Collins M, Hegarty K, Priddle S, Smith J et al Axial elongation following prolonged near work in myopes and emmetropes. Br J Ophthalmol. 2010;95: 652–656. 10.1136/bjo.2010.180323 20829316

[8] Read SA, Collins MJ, Sander BP. Human optical axial length and defocus. Invest Ophthalmol Vis Sci. 2010;51: 6262–6269. 10.1167/iovs.10-5457 20592235

[9] P¨arssinen O. Anisometropia and changes in anisometropia in school myopia. Optometry and vision Science. 1990;67: 256–259. 2188189 10.1097/00006324-199004000-00005

[10] Yamashita T, Watanabe S, Ohba N. A longitudinal study of cycloplegic refraction in a cohort of 350 Japanese schoolchildren. Anisometropia. Ophthalmic and Physiological Optics. 1999;19: 30–33. 10615436 10.1046/j.1475-1313.1998.00407.x

[11] Huynh SC, Wang XY, Ip J, Robaei D, Kifley A, Rose KA, et al Prevalence and associations of anisometropia and aniso-astigmatism in a population based sample of 6 year old children. Br J Ophthalmol. 2006;90: 597–601. 10.1136/bjo.2005.083154 16622090 PMC1857062

[12] Tong L, Chan Y, Gazzard G, Tan D, Saw S. Longitudinal Study of Anisometropia in Singaporean School Children. Invest Ophthalmol Vis Sci. 2006;47: 3247–3252. 10.1167/iovs.05-0906 16877388

[13] Giordano L, Friedman D, Repka M, Katz J, Ibironke J, Hawes P et al Prevalence of refractive error among preschool children in an urban population: the Baltimore Pediatric Eye Disease Study. Ophthalmology. 2009;116: 739–746. 10.1016/j.ophtha.2008.12.030 19243832 PMC2680482

[14] Borchert M, Tarczy-Hornoch K, Cotter S, Liu N, Azen S, Varma R. Anisometropia in Hispanic and African American infants and young children the multi-ethnic pediatric eye disease study. Ophthalmology. 2010;117: 148–153. 10.1016/j.ophtha.2009.06.008 19818509 PMC2814917

[15] Yekta A, Fotouhi A, Hashemi H, Dehghani C, Ostadimoghaddam H, Heravian J, et al Prevalence of refractive errors among schoolchildren in Shiraz, Iran. Clin Experiment Ophthalmol. 2010; 38: 242–248. 10.1111/j.1442-9071.2010.02247.x 20447119

[16] Deng L. Gwiazda J. Anisometropia in Children from Infancy to 15 Years. Investigative Opthalmology & Visual Science. 2012;53: 3782–3786. 10.1167/iovs.11-8727 PMC3390183 22589429

[17] Afsari S, Rose K, Gole G, Philip K, Leone J, French A, et al Prevalence of anisometropia and its association with refractive error and amblyopia in preschool children. Br J Ophthalmol. 2013;97: 1095–1099. 10.1136/bjophthalmol-2012-302637 23613508

[18] O'Donoghue L, McClelland J, Logan N, Rudnicka A, Owen C, Saunders K. Profile of anisometropia and aniso-astigmatism in children: prevalence and association with age, ocular biometric measures, and refractive status. Invest Ophthalmol Vis Sci. 2013;54: 602–608. 10.1167/iovs.12-11066 23233258

[19] Hu YY, Wu JF, Lu TL, Wu H, Sun W, Guo da D et al Prevalence and associations of anisometropia in Children. Invest Ophthalmol Vis Sci. 2016;57: 979–988. 10.1167/iovs.15-18647 26962694

[20] Weale R. On the age-related prevalence of anisometropia. Ophthalmic Res. 2002;34: 389–392. 12483028 10.1159/000067040

[21] Sherwin JC, Reacher MH, Keogh RH, Khawaja AP, Mackey DA, Foster PJ. The association between time spent outdoors and myopia in children and adolescents: a systematic review and meta-analysis. Ophthalmology. 2012;119: 2141–2151. 10.1016/j.ophtha.2012.04.020 22809757

[22] Huang HM, Chang DS, Wu PC. The Association between Near Work Activities and Myopia in Children-A Systematic Review and Meta-Analysis. PLOS One. 2015;10:e0140419 10.1371/journal.pone.0140419 26485393 PMC4618477

[23] Tsai DC, Lin LJ, Huang N, Hsu CC, Chen SY, Chiu AW, Liu CJ. Study design, rationale, and methods for a population-based study of myopia in schoolchildren: the Myopia Investigation study in Taipei. Clin Experiment Ophthalmol. 2015;43: 612–620 10.1111/ceo.12532 25881723

[24] Ojaimi E, Rose KA, Smith W., Morgan IG, Martin FJ, Mitchell P. Methods for a population-based study of myopia and other eye conditions in school children: the Sydney Myopia Study. Ophthalmic Epidemiol. 2005;12: 59–69. 10.1080/09286580490921296 15848921

[25] Qin XJ, Margrain TH, To CH, Bromham N, Guggenheim JA. Anisometropia is independently associated with both spherical and cylindrical ametropia. Invest Ophthalmol Vis Sci. 2005;46: 4024–4031. 10.1167/iovs.05-0120 16249476

[26] Huang HM, Chang DS, Wu PC. The Association between Near Work Activities and Myopia in Children-A Systematic Review and Meta-Analysis. PLoS One. 2015;201;10:e0140419. 10.1371/journal.pone.0140419 PMC4618477 26485393

[27] Mutti DO, M itchell GL, Moeschberger ML, Jones LA, Zadnik K. Parental myopia, near work, school achievement and children’s refractive error. Invest Ophthalmol Vis Sci. 2002; 43: 3633–3640. 12454029

[28] Saw SM, Chua WH, Hong CY, Wu HM, Chan WY, Chia KS, et al Near work in early-onset myopia. Invest Ophthalmol Vis Sci. 2002; 43: 332–339. 11818374

[29] Saw SM, Zhang MZ, Hong RZ, Fu ZF, Pang MH, Tan DT. Near-work activity, night-lights, and myopia in the Singapore-China study. Arch Ophthalmol. 2002; 120: 620–627. 12003612 10.1001/archopht.120.5.620

[30] Hsu CC, Huang N, Lin PY, Tsai DC, Tsai CY, Woung LC, et al Prevalence and risk factors for myopia in second-grade primary school children in Taipei: A population-based study. J Chin Med Assoc. 2016 6 24. pii: S1726-4901(16)30078-8. 10.1016/j.jcma.2016.02.011 27349942

[31] Lin Z, Vasudevan B, Liang Y, Zhang Y, Zhao S, Yang X et al Nearwork-induced transient myopia (NITM) in anisometropia. Ophthalmic Physiol Opt. 2013;33: 311–317. 10.1111/opo.12049 23662963

[32] Lewis HA, Kao CY, Sinnott LT, Bailey MD. Changes in ciliary muscle thickness during accommodation in children. Optom Vis Sci. 2012;89: 727–737. 10.1097/OPX.0b013e318253de7e 22504329 PMC3348375

[33] Bailey MD, Sinnott LT, Mutti DO. Ciliary body thickness and refractive error in children. Invest Ophthalmol Vis Sci. 2008; 49: 4353–4360. 10.1167/iovs.08-2008 18566470 PMC2994597

[34] Kuchem MK, Sinnott LT, Kao CY, Bailey MD. Ciliary muscle thickness in anisometropia. Optom Vis Sci. 2013;90: 1312–1320. 10.1097/OPX.0000000000000070 24100479 PMC3985092

[35] Guzowski M, Fraser-Bell S, Rochtchina E, Wang JJ, Mitchell P. Asymmetric refraction in an older population: the Blue Mountains Eye Study. Am J Ophthalmol. 2003;136: 551–553. 12967817 10.1016/s0002-9394(03)00246-0

