# Supplementary material for: Prevalence and association of refractive anisometropia with near work habits among young schoolchildren: The evidence from a population-based study
Source: PLoS One. 2017 Mar 8;12(3):e0173519. doi: 10.1371/journal.pone.0173519 (PMC5342266; doi:10.1371/journal.pone.0173519)
Supplement: S1 Table — (DOC) [file pone.0173519.s001.doc]

**S1 Table**. **Survey questions and responses in the questionnaire.**

| **Demographics and medical history of child** |
| --- |
| - name, gender, contact information, main caregiver |
| - Does your child have history of premature birth (□no, □yes), asthma (□no, □yes), or atopic dermatitis (□no, □yes)? |
| - Does your child have amblyopia? □no, □yes   - If yes, does he/she receive any treatment? □no, □yes |
| - Does your child have strabismus? □no, □yes   - If yes, does he/she receive any treatment? □no, □yes |
| - Does your child have myopia? □no, □yes - If yes, the refraction status: □less than -3.0D, □-3.0D or higher, □unknown - If yes, does he/she wear glasses? □no, □yes - If yes, does he/she receive other treatment (e.g., corneal reshaping contact lens, cycloplegic eye drops)? □no, □yes |
| - When did your child start learning to write, paint, play on the computer, or perform other near work? □< 3 years old, □≥ 3 but <4 years old, □≥ 4 but <5 years old, □≥ 5 but <6 years old □≥ 6 years old |
| **Parents’ information** |
| - Education level (father/mother): □illiterate, □primary school, □junior high school, □senior high school/vocational school, □junior college, □university, □graduate school, □unknown |
| - Occupation (father/mother): □no, □work for government/school (soldiers, police, public servants, and teachers), □laborer, □merchant, □self-employment, □agriculture, forestry, fishing, and animal husbandry, □househusband, □others­­_______ |
| - Do the child’s parents have myopia? (father/mother) □no, □yes, □unknown - If yes, the refraction status is:□less than -6.0D, □-6.0D or higher, □unknown |
| **Near work activities** |
| - What is the distance from the eye to the object when your child writes, paints, plays on the computer, or performs other near work? □≥30cm, □< 30cm, □unknown |
| - Over the past year, did your child use cellphones, computers, or tablet personal computers? □no, □yes - If yes, how old was he/she when he/she started to use these products? □< 3 years old, □≥ 3 but <4 years old, □≥ 4 but <5 years old, □≥ 5 but <6 years old □≥ 6 years old - If yes, how much time a day did he/she spend using these products? □< 1 hour, □≥ 1 but < 2 hours, □≥ 2 but< 4 hours, □≥ 4 hours |
| - Over the past year, how much time a day did your child spend watching television? □< 1 hour, □≥ 1 but < 2 hours, □≥ 2 but< 4 hours, □≥ 4 hours |
| - What is the distance from the eye to the television when your child watches it?□< 1 meter, □≥ 1 but <2 meters, □≥ 2 but <3 meters, □≥ 3 meters |
| - Over the past year, how much time a day did your child spend on writing, painting, playing on the computer, or performing other near work? □< 1 hour, □≥ 1 but < 2 hours, □≥ 2 but< 4 hours, □≥ 4 hours |
| - Does your child take a rest for over 10 minutes after doing near work for 30 minutes? □no, □yes, □unknown |
| - Does your child use table lamps when he/she reads or writes? □no, □yes, □unknown |
| - Does your child read while lying down? □never, □seldom, □sometimes, □usually, □always, □unknown |
| **After-school tutorial program** |
| - Did your child ever participate in an after-school program/cram school? □no, □yes   - If yes, he/she has attended a program for □< 1 year, □≥ 1 but < 2 years, □≥ 2 but < 3 years, □≥ 3 years |
| - How much time a week did your child spend participating in the after-school programs/cram schools? □< 5 hours, □≥ 5 but <10 hours, □≥ 10 hours |
| **Outdoor activities** |
| - After school, how much time a day on weekdays does your child spend on outdoor activities (e.g., sports, playing balls, waking in the park)? □none, □< 30 minutes, □≥30 minutes but < 1 hour, □≥ 1 but < 2 hours, □≥ 2 but < 4 hours, □≥ 4 hours |
| - How much time a day on the weekend does your child spend on outdoor activities (e.g., sports, playing balls, waking in the park)? □none, □< 30 minutes, □≥30 minutes but < 1 hour, □≥ 1 but < 2 hours, □≥ 2 but < 4 hours, □≥ 4 hours |
| **Eye checks** |
| - Did you have your child’s eyes examined by an ophthalmologist over the past year? □no, □yes, □unknown |
| **Knowledge about high myopia** |
| - As per your knowledge, high myopia means refraction power higher than: □-3.0D, □-4.0D, □-5.0D, □-6.0D |
| - Which kinds of ophthalmic complications are associated with high myopia? (you may select multiple responses) □glaucoma, □maculopathy, □night blindness, □loss of vision, □cataract (early onset), □retinal detachment, □unknown, □others_________________ |
|  |
